# Ampicillin Plus Ceftriaxone Combined Therapy for *Enterococcus faecalis* Infective Endocarditis in OPAT

**DOI:** 10.3390/jcm11010007

**Published:** 2021-12-21

**Authors:** Laura Herrera-Hidalgo, Jose Manuel Lomas-Cabezas, Luis Eduardo López-Cortés, Rafael Luque-Márquez, Luis Fernando López-Cortés, Francisco J. Martínez-Marcos, Javier de la Torre-Lima, Antonio Plata-Ciézar, Carmen Hidalgo-Tenorio, Maria Victoria García-López, David Vinuesa, Alicia Gutiérrez-Valencia, Maria Victoria Gil-Navarro, Arístides De Alarcón

**Affiliations:** 1 Unidad de Farmacia, Hospital Universitario Virgen del Rocío/CSIC/Instituto de Biomedicina de Sevilla (IBiS), 41013 Seville, Spain; lauraherrerahidalgo@gmail.com; 2 Unidad Clínica de Enfermedades Infecciosas, Microbiología y Medicina Preventiva, Hospital Universitario Virgen del Rocío/CSIC/Instituto de Biomedicina de Sevilla (IBiS), 41013 Seville, Spain; jlomascabezas@yahoo.es (J.M.L.-C.); rafael.luque.sspa@juntadeandalucia.es (R.L.-M.); luisfernando@lopezcortes.net (L.F.L.-C.); alicia.gutierrez.valencia@gmail.com (A.G.-V.); aa2406ge@yahoo.es (A.D.A.); 3 Unidad Clínica de Enfermedades Infecciosas, Microbiología y Medicina Preventiva, Hospital Universitario Virgen Macarena/CSIC/Instituto de Biomedicina de Sevilla (IBiS), 41009 Seville, Spain; luiselopezcortes@gmail.com; 4 Unidad de Enfermedades Infecciosas, Hospital Juan Ramón Jiménez, 21005 Huelva, Spain; fcojmtz@telefonica.net; 5 Unidad de Enfermedades Infecciosas, Servicio de Medicina Interna, Hospital Costa del Sol, 29603 Marbella, Spain; jtorrel@gmail.com; 6 Servicio de Enfermedades Infecciosas, Hospital Regional Universitario de Malaga, 29010 Málaga, Spain; nonispc@hotmail.com; 7 Servicio de Enfermedades Infecciosas, Hospital Universitario Virgen de las Nieves, 18014 Granada, Spain; chidalgo72@gmail.com; 8 Servicio de Microbiología, Hospital Universitario Virgen de la Victoria, 29010 Málaga, Spain; mav.garcia@hotmail.com; 9 Servicio de Enfermedades Infecciosas, Hospital Universitario San Cecilio, 18016 Granada, Spain; vinudav@yahoo.es

**Keywords:** *Enterococcus faecalis*, infective endocarditis, ampicillin, ceftriaxone, outpatient parenteral antibiotic treatment

## Abstract

Ampicillin plus ceftriaxone (AC) is a well-recognized inpatient regimen for *Enterococcus faecalis* infective endocarditis (IE). In this regimen, ceftriaxone is usually administered 2 g every 2 h (AC12). The administration of AC in outpatient parenteral antibiotic treatment (OPAT) programs is challenging because multiple daily doses are required. AC regimens useful for OPAT programs include once-daily high-dose administration of ceftriaxone (AC24) or AC co-diluted and jointly administered in bolus every 4 h (ACjoined). In this retrospective analysis of prospectively collected cases, we aimed to assess the clinical effectivity and safety of three AC regimens for the treatment of *E. faecalis* IE. Fifty-nine patients were treated with AC combinations (AC12 *n* = 32, AC24 *n* = 17, and ACjoined *n* = 10). Six relapses occurred in the whole cohort: five (29.4%) treated with AC24 regimen and one (10.0%) with ACjoined. Patients were cured in 30 (93.3%), 16 (94.1%), and eight (80.0%) cases in the AC12, AC24 and ACjoined groups, respectively. Unplanned readmission occurred in eight (25.0%), six (35.3%), and two (20.0%) patients in the AC12, AC24 and ACjoined groups, respectively. The outcome of patients with *E. faecalis* IE treated with AC in OPAT programs relies on an optimization of the delivery of the combination. AC24 exhibit an unexpected rate of failures, however, ACjoined might be an effective alternative which clinical results should corroborate in further studies.

## 1. Introduction

Enterococcal endocarditis is a severe disease with incidence that has significantly increased in the last decades [1]. First-line antibiotic regimens recommended by international guidelines comprise the combination of a high-dose penicillin (ampicillin, amoxicillin or benzylpenicillin) plus a synergistic agent (ceftriaxone or gentamycin) for 4–6 weeks [2,3]. Outpatient parenteral antibiotic treatment (OPAT) is an advantageous alternative to long inpatient treatments, and the inclusion of patients with infective endocarditis (IE) has been globally endorsed [4,5]. The optimal treatment of *E. faecalis* IE in the outpatient setting has not yet been established, which is mostly attributed to the challenging adaptation of multi-dose fist line treatments in this scenario [6].

Ampicillin 2 g every 4 h plus ceftriaxone 2 g every 12 h combined therapy (AC12) is the preferred inpatient regimen, since it shows a lower rate of adverse effects than the regimens based on the combination of ampicillin plus gentamycin [7]. The major concerns regarding AC12 administration delivered via OPAT are ampicillin solution stability and the need for a twice-daily administration of ceftriaxone. Twice-daily visitation increases the resource consumption and prevents its global implantation in OPAT programs. Ampicillin administration through an electronic pump is a reliable option since a recent study clarified the existing doubts regarding stability in an OPAT scenario [8]. Different options should be considered with ceftriaxone administration: (i) maintaining the inpatient regimen, which implies twice-daily nurse visitation, (ii) grouping the daily dose in a single 4 g administration, (iii) dilute together with ampicillin for a joint administration of both antibiotics through an electronic pump (programmed to release 2 g of ampicillin and 666 mg of ceftriaxone 4-hourly), since the stability of the combined solution has been recently proved [9]. The clinical experience with the regimen that included a single daily-dose of ceftriaxone is only four cases reported with favourable results [10] and null with the joined administration.

The original ceftriaxone regimen (2 g every 12 h) was design to theoretically maintain ceftriaxone plasma concentration over the stablished synergy threshold (5 µg/mL) between both antibiotics [11,12]. Recently, it has been demonstrated that both the original regimen and 4 g single daily-dose failed to maintain this concentration [13]. The pharmacokinetics of ceftriaxone administered 666 mg every 4 h has not been studied, but it could be hypothesized that more frequent administration could help to achieve this goal.

We aimed to assess the clinical effectivity and safety of these three AC regimens for the treatment of *E. faecalis* infective endocarditis.

## 2. Materials and Methods

We performed a retrospective analysis of prospectively collected cases from a cohort attended at two tertiary hospitals between 2005 and 2021. Adult patients with definite or possible endocarditis, according to the modified Duke criteria [14], treated with an AC regimen for enterococcal endocarditis and recorded in pre-existing endocarditis and OPAT databases were selected. Length of therapy according with international guidelines was required [2,3]. Antimicrobial treatment and patient inclusion criteria in an OPAT program were settled on by a multidisciplinary team. The OPAT program included daily visitation by the nurse team for drug administration and clinical care, and weekly reviews by an infectious diseases physician.

Medical records were recorded prospectively to gather information including demographic data, episode of endocarditis, treatment management and clinical outcomes. All patients were initially treated with the inpatient AC regimen (ampicillin 2 g every 4 h plus ceftriaxone 2 g every 12 h) until clinical and stabilization. AC continuation treatment was classified into three groups according to ceftriaxone administration: (i) patients who continued hospitalized with ceftriaxone 2 g every 12 h (AC12 group); (ii) patients included in the OPAT program treated with ceftriaxone 4 g every 24 h (AC24 group); (iii) patients included in the OPAT program treated with ceftriaxone diluted jointly with ampicillin and administered in pulses every 4 h (2 g de ampicillin + 666 mg of ceftriaxone every 4 h) (ACjoined group). Patients included between 2005 and the start date of our OPAT program (2012) were treated with the AC12 regimen. Ampicillin dose regimen was 2 g every 4 h, except for renal impairment adjustments.

Infective endocarditis was classified depending on the location (right vs left side) and the valve or cardiac device involved. The Charlson comorbidity index was used to grade overall morbidity at the time of diagnosis [15]. Minimum and maximum follow-up were established at 6 and 12 months, respectively, after completion of the antibiotic treatment, with periodic clinical reviews and follow-up blood cultures to ensure definitive microbiological cure. Outcomes assessed during the follow-up period were: (i) relapse, defined as positive blood cultures caused by the same microorganism as the initial episode; (ii) overall mortality, defined as death from any cause; (iii) endocarditis-related mortality, defined as death derived from the infection or its sequalae; and (iv) adverse events related with AC treatment. For those relapses observed after six months of treatment stop-date, pulsed field gel electrophoresis (PFGE) was used to distinguish the strains [16]. STROBE guidelines for reporting observational studies were followed [17]. The study was approved by the Ethics Committee for Clinical Research of Seville (2396-N-21).

The statistical analysis was performed using R-studio and SPSS version 28.0 (SPSS Inc., Chicago, IL, USA). Categorical variables were summarized as percentages. Continuous variables were summarized as median and interquartile range (IQR). Quantitative variables were compared using a Kruskal–Wallis test and categorical variables were compared using the Chi-Square test. A two-sided *p* < 0.05 was considered statistically significant.

## 3. Results

Fifty-nine patients with *E. faecalis* IE were treated with AC combinations. The treatment was AC12, AC24 and ACjoined in 32 (54.2%), 17 (28.8%) and 10 (17.0%) patients, respectively (Table 1). Comorbidities did not differ significantly between the three groups, with the exception of colorectal disease, which was more frequent in the AC24 group (52.9%) respect to the AC12 and ACjoined groups (12.5% and 30%, respectively) (*p* = 0.01). A trend towards (*p* = 0.07) a worse Charlson score has been observed in the OPAT regimens, although it was not statistically significant. The median duration of AC treatment was 42 days and was similar between the three groups. Regarding the OPAT regimens (AC24 and ACjoined), the median duration of inpatient antibiotic treatment prior to OPAT was 26.6 ± 9.3 and 16.2 ± 8.3 days in the AC24 and ACjoined groups, respectively (*p* = 0.007).

Forty-three (72.9%) were classified as definite IE and 16 (27.1%) as possible IE in accordance with the modified Duke criteria. Fourteen patients (23.7%) presented more than one structure involved in the infection (Table 2). Prosthetic valve IE was diagnosed in seven (21.9%), four (23.5%) and six (60.0%) patients in AC12, AC24 and ACjoined (*p* = 0.025), respectively. Twenty-five percent (*n* = 8) of the surgery indicated was never performed, two in the AC12 group (10% of indicated surgery), four in the AC24 group (40% of indicated surgery) and two in the ACjoined group (100% of indicated surgery).

Six relapses occurred in the complete whole: five (29.4%) patients had been treated with AC24 regimen and one (10.0%) patient with ACjoined. Relapsed episodes are detailed in Table 3. Among the patients receiving a complete in-patient treatment (AC12), 30 (93.3%) of them were cured and two (6.3%) died. Unplanned readmission after hospital discharge occurred in eight (25.0%) of them during the follow-up period (cardiac insufficiency *n* = 3; deep vein thrombosis *n* = 1; cerebral hemorrhage *n* = 1, decreased level of consciousness *n* = 1; cardiorespiratory arrest *n* = 1; and late hypersensitivity to antibiotics and tachycardia *n* = 1). Patients included in OPAT (*n* = 27, 45.8%) were treated with AC24 or ACjoined as continuation regimens. Out of the seventeen patients treated with AC24, 16 (94.1%) were cured and one (5.9%) died. Six (35.3%) of them required unplanned hospitalization, in four (23.5%) cases due to relapses, in one due to cardiac insufficiency, and one operated patient with a new IE episode caused by another microorganism. The mean length of inpatient treatment in AC24 relapsed episodes was 16.8 ± 4.0 days, whilst the not-relapsed group was 30.6 ± 7.6 days (*p* = 0.003). Ten patients received ACjoined as continuation regimen. Among them, eight (80.0%) were cured and two (20.0%) of them died. In both cases surgery had been indicated but not performed. In this cohort unplanned readmission occurred in two (20.0%) patients (cerebral toxoplasmosis *n* = 1; and pseudomembranous colitis *n* = 1). One patient in the ACjoined group completed the last 7 days of treatment with oral therapy due to a vascular access complication. Seven patients experienced side effects related to AC treatment, six (85.0%) belonging to the AC12 group and one (15.0%) to the ACjoined group (*p* = 0.152) (Table 2).

## 4. Discussion

This is the first study showing how to adapt AC combination to the outpatient setting and comparing clinical results of two AC dose regimens for *E. faecalis* infective endocarditis in OPAT programs and the conventional inpatient AC regimen. This double β-lactam combination has been endorsed by preclinical data proving its synergistic activity [11,12] and clinical good results in extensive cohorts [6], being nowadays recognized as an alternative to ampicillin plus gentamycin regimens by international guidelines for *E. faecalis* infective endocarditis as first line therapy [2,3].

AC administration at home requires some adjustment given the difficulty of delivering a twice-daily ceftriaxone regimen. We proposed two new AC schemes: AC24 and ACjoined. AC24 comprises the administration of a single-daily dose of 4 g of ceftriaxone. This regimen allows once-daily nurse visitation, but it might implicate drug insufficient concentrations to maintain the synergistic activity 8 h after the dose [13]. In this cohort, we found a greater number of relapses (29.4%) in patients treated with the AC24 continuation regimen than the original AC12 (0.0%). This high number of relapses could be influenced by the large number of patients with prosthetic valve infection and device-related infections, the poor initial condition and the lack of surgical management despite being indicated as risk factors for treatment failure [19]. Additionally, time until discharge was shorter than 21 days in all relapses, which raises the question that AC24 might be insufficient due to low ceftriaxone concentrations after 18 h with this dosing regimen [13], and suggesting that the clinical success might be attributed to a longer inpatient treatment under the AC12 regimen for at least three weeks. Despite the possible explanations detailed, caution is required when treating enterococcal endocarditis with the AC24 scheme in OPAT programs.

Continuous infusion of benzylpenicillin plus ceftriaxone (BC) has been recently explored as a double β-lactam combination in the outpatient setting [20,21]. This combination raises some concerns that must be addressed. Firstly, it has been assumed that the synergistic activity exhibited by AC [14,15] is preserved in BC combination [20]. However, recent studies have shown poor correlation between AC and BC synergistic activity and lower rates of this interaction with BC in *E. faecalis* clinical strains [21,22]. Secondly, ceftriaxone dose regimen adjustment for OPAT entails the same difficulty shown in the AC regimen. In these studies, ceftriaxone dosing was highly variable and included regimens unable to provide synergy [13], such as, for example, 2 g daily dose [20]. Finally, clinical outcomes from *E. faecalis* IE treated with BC were controversial. One study exhibited a low relapse rate (5%), whereas 35% of the patients continued with long-term suppressive antibiotic treatment after BC completion [20]. Another study showed high rates of unplanned readmissions (30%), although rates of relapsed bacteremia (5%) were low [21]. Overall, prior to recommending BC therapy further investigation is still required, not only for efficacy and dosing design, but also for the molecular basis of the combination.

As an alternative, we present an AC scheme suitable for the outpatient setting with promising results. ACjoined consist in the co-dilution of the daily dose of both antibiotics in the same solution and the administration together through an electronic pump in bolus every 4 h. Initially, this option was avoided due to the absent of stability data. Nevertheless, once drug stability had been proved in similar conditions than the OPAT program [9], ACjoined was used as continuation treatment for patients with enterococcal endocarditis. In our population treated with ACjoined, only one patient relapsed (10%) in spite of none of the indicated surgery being performed and the elevated number of prosthetic endocarditis included (60.0%). However, despite these promising results, further investigation is required to consolidate the ACjoined scheme as the best option for the treatment of *E. faecalis* IE in OPAT programs.

Other therapeutic alternatives for *E. faecalis* IE outpatient continuation regimen have been discussed [6]. Dalbavancin and teicoplanin are antibiotics with long half-life which are easily included in OPAT programs. The experience with these antibiotics in this scenario is limited but promising, especially for teicoplanin [23,24]. Another option, recently endorsed by the results of a large clinical trial, for the continuation regimen of *E. faecalis* infective endocarditis is oral treatment, although, *E. faecalis* episodes included were scarce [25].

The interpretation of our study results is bounded by its retrospective design and the low number of patients included. Furthermore, follow-up period varied between 6 and 12 months, which could be insufficient for later relapses. Despite of these limitations, our study paves the way for further investigation regarding the administration of fist line regimens for enterococcal endocarditis in the outpatient setting.

## 5. Conclusions

The outcome of patients with *E. faecalis* IE treated with AC in OPAT programs relies on an optimization of the delivery of the combination. AC24 exhibit an unexpected rate of failures, but ACjoined might be an effective alternative which clinical results should corroborate in further studies.

## Figures and Tables

**Table 1:** Baseline characteristics.

Baseline Characteristics	Overall (*n* = 59)	Treatment			*p*
		AC12 (*n* = 32)	AC24 (*n* = 17)	ACjoined (*n* = 10)	
**Age (median (IQR))**	**68 (59–77)**	64 (57–73)	73 (60–80)	73 (59–77)	0.127
Male gender	37 (62.7)	18 (56.3)	13 (76.5)	6 (60.0)	0.372
Charlson score (median (IQR))	4 (3–5)	3 (2–5)	5 (3.5–5.5)	5 (3.75–5.5)	0.07
Comorbidities					
Hypertension	32 (54.2)	16 (50.0)	9 (52.9)	7 (70.0)	0.537
Diabetes mellitus	17 (28.8)	11 (34.4)	3 (17.6)	3 (30.0)	0.467
Hyperlipidaemia	20 (33.9)	9 (28.1)	7 (41.2)	4 (40.0)	0.593
Chronic renal failure	13 (22.0)	9 (28.1)	3 (17.6)	1 (10.0)	0.422
Colorectal disease	16 (27.1)	4 (12.5)	9 (52.9)	3 (30.0)	**0.010**
Chronic obstructive pulmonary disease	8 (13.6)	6 (18.8)	2 (11.8)	0 (0.0)	0.309
Cancer	9 (15.3)	5 (15.6)	4 (23.5)	0 (0.0)	0.259
Peripheral vascular disease	9 (15.3)	2 (6.3)	4 (23.5)	3 (30.0)	0.101
Liver disease	6 (10.2)	5 (15.6)	1 (5.9)	0 (0.0)	0.284
Previous cerebrovascular accident	5 (8.5)	2 (6.3)	2 (11.8)	1 (10.0)	0.790
Previous IE episode	3 (5.1)	0 (0.0)	2 (11.8)	1 (10.0)	0.094
Prosthetic valve/Pacemaker carrier (involved or not) *	22 (37.3)	9 (28.1)	7 (41.2)	6 (60.0)	0.177
Prosthetic valve location (involved or not)					
Prosthetic aortic valve	20 (33.9)	7 (21.9)	7 (41.2)	6 (60.0)	0.148
Prosthetic mitral valve	4 (6.8)	2 (6.3)	1 (5.9)	1 (10.0)	0.925
Prosthetic tricuspid valve	1 (1.7)	1 (3.1)	0 (0.0)	0 (0.0)	0.601
Type of prosthesis (involved or not) *					
Valvular prosthesis	17 (28.8)	7 (21.9)	5 (29.4)	5 (50.00)	0.555
Pacemaker	4 (6.8)	2 (6.3)	2 (11.8)	0 (0.0)	
TAVI	3 (5.1)	0 (0.0)	2 (11.8)	1 (10.00)	

Bold indicates statistical significance, IQR = Interquartile range, IE = Infective endocarditis, TAVI = Transaortic valve implantation, * Some patients carried more than one prosthetic valve or a prosthetic valve and a pacemaker.

**Table 2:** Infection-related characteristics and clinical outcomes.

Endocarditis Characteristics	Overall (*n* = 59)	Treatment			*p*
		AC12 (*n* = 32)	AC24 (*n* = 17)	ACjoined (*n* = 10)	
**Type of endocarditis**					0.515
Left-side IE	53 (89.8)	29 (90.6)	16 (94.1)	8 (80.0)	
Right-side IE	2 (3.4)	1 (3.1)	0 (0.0)	1 (10.0)	
Left and right-side IE	1 (1.7)	1 (3.1)	0 (0.0)	0 (0.0)	
Other or unknown	3 (5.1)	1 (3.1)	1 (5.9)	1 (10.0)	
Native valve IE	39 (66.1)	25 (78.1)	11 (64.7)	3 (30.0)	**0.019**
Early prosthetic valve IE (<1 year)	7 (11.9)	2 (6.3)	1 (5.9)	4 (40.0)	**0.010**
Late prosthetic valve IE (>1 year)	10 (16.9)	5 (15.6)	3 (17.6)	2 (20.0)	0.946
Cardiac device-related IE	3 (5.1)	0 (0.0)	2 (11.8)	1 (10.0)	0.151
Valve involvement					
Aortic valve	28 (47.5)	16 (50.0)	8 (47.1)	4 (40.0)	0.858
Mitral valve	14 (23.7)	8 (25.0)	4 (23.5)	2 (20.0)	0.948
Mitral and aortic valves	11 (18.6)	5 (15.6)	4 (23.5)	2 (20.0)	0.790
Mitral and tricuspid valves	1 (1.7)	1 (3.1)	0 (0.0)	0 (0.0)	0.651
IVC and tricuspid valve	2 (3.4)	1 (3.1)	0 (0.0)	1 (10.0)	0.380
Other or unknown	3 (5.1)	1 (3.1)	1 (5.9)	1 (10.0)	0.678
Cardiac Surgery					
Cardiac surgery indicated *	32 (54.2)	20 (62.5)	10 (58.8)	2 (20.0)	**0.049**
Cardiac surgery performed (% of indicated)	24 (75.0)	18 (90.0)	6 (60.0)	0 (0.0)	**0.008**
**Clinical outcomes**					
Relapses	6 (10.2)	0 (0.0)	5 (29.4)	1 (10.0)	**0.005**
Side effects related to AC	7 (11.9)	6 (18.8)	0 (0.0)	1 (10.0)	0.152
Unplanned readmission	17 (28.8)	9 (28.1)	6 (35.3)	2 (20.0)	0.693
Readmission unrelated to IE	8 (13.6)	5 (15.6)	2 (11.8)	2 (20.0)	0.260
Readmission related to IE	9 (15.3)	4 (12.5)	5 (29.4)	0 (0.0)	
Final outcome					
Cured	54 (91.5)	30 (93.8)	16 (94.1)	8 (80.0)	0.177
Death unrelated to IE	2 (3.4)	1 (3.1)	1 (5.9)	0 (0.0)	
Death related to IE	3 (5.1)	1 (3.1)	0 (0.0)	2 (20.0)	

Bold indicates statistical significance, IQR = Interquartile range, IE = Infective endocarditis, AC = Ampicillin plus ceftriaxone treatment, IVC = Interventricular communication, * Surgical indications were evaluated according to the American Association for Thoracic Surgery (AATS) guidelines [18].

**Table 3:** Description of relapsed episodes.

Age	CS ^#^	Type IE	AC Group	Surgery (i/p) *	Commentaries	Outcome (Follow-Up)
87	6	Early prosthetic aortic valve IE in a patient with a pacemaker	AC24	No/No	Pacemaker replacement (pacemaker wire cultures negative) and treatment with daptomycin followed by dalbavancin (9 weeks)	Cure (1 year)
60	1	Native mitral valve IE in a patient with a prosthetic aortic valve	AC24	No/No	Treatment with teicoplanin (8 weeks)	Cure (1 year)
68	3	Early prosthetic mitral and aortic valve IE in a patient with a pacemaker	AC24	No/No	Treatment with ampicillin (6 weeks) plus gentamicin (2 weeks).	Cure (1 year)
73	4	Native mitral valve IE	AC24	No/No	Treatment with ampicillin (6 weeks) plus gentamicin (2 weeks). Second relapse and treatment with ampicillin plus teicoplanin followed by amoxicillin plus moxifloxacin (10 weeks)	Cure (1 year)
81	4	Native mitral and aortic valve IE with pseudoaneurysm of the radial and femoral arteries	AC24	No/No	Treatment with ampicillin plus ceftriaxone (6 weeks) and cardiac surgery	Cure (1 year)
88	5	Native mitral and prosthetic aortic valve complicated IE with pseudoaneurysm of the mitral-aortic intervalvular fibrosa	AC joined	Yes/No	Relapsed and death	Death (2 months)

^#^ CS: Charlson score, * (i/p): Surgery indicated/Surgery performed. Surgical indications were evaluated according to the American Association for Thoracic Surgery (AATS) guidelines [18].
